# Neural Network Technology-Based Optimization Framework of Financial and Management Accounting Model

**DOI:** 10.1155/2022/4991244

**Published:** 2022-05-31

**Authors:** Yan Zeng

**Affiliations:** Wuhan College, Wuhan 430000, China

## Abstract

Traditional financial accounting has gradually evolved into management accounting in order to adapt to changing times and developments. To avoid being obliterated by the times, accountants must gradually improve their professional and comprehensive abilities in order to create greater value for businesses in the AI (Artificial Intelligence) era. This article presents an AI-based financial management optimization design and proposes an AI-based accounts receivable management optimization framework based on the existing information system. A typical financial distress early-warning model is built using the BPNN (BP Neural Network) model, and the training samples of listed companies' financial data are processed iteratively using the neural network algorithm to realize the visual modeling of the object-oriented neural network and learn the training samples. Finally, the network's ability to provide early warning is put to the test. The results show that BPNN's prediction accuracy is significantly higher than that of other types, especially after years of data, with prediction results exceeding 90%. The results show that the BPNN-based financial early-warning method is feasible.

## 1. Introduction

Accounting is the statistical analysis of an enterprise's financial data using professional recording methods in accordance with the accounting financial system's basic principles and the use of financial data to reflect a company's financial transactions and business information at a given point in time. Accounting is a management information system that employs systematic methods to analyze, evaluate, and manage the financial and economic data generated by an enterprise's business activities and assists management in providing supporting data for management decisions and operations. With the advancement of economic levels, generating more value in order to achieve sustainable development has become a problem that every business will face. The gradual implementation of AI (Artificial Intelligence) technology [1, 2] has a significant impact on social development and accounting work, with some traditional financial personnel potentially losing their jobs. In light of this, financial accounting must keep up with the times and strive to transform into management accounting in order to adapt to the transformation and development of the times. AI has accelerated the transformation and promotion of accounting work in the field of accounting. Financial robots can independently identify financial data, making large amounts of data processing simple and quick. At the same time, it imposes more stringent requirements on businesses.

In recent years, some issues in the company's economic activities, financial management, and high-risk fields have been exposed, whether through external audit or internal special inspection. It is necessary to research and develop perfect financial audit system functions and establish a unified financial audit management method and mode, in order to find and solve problems as soon as possible. To achieve centralized financial system management, Eckles et al. proposed that each member unit's financial business be concentrated in the financial sharing service center or rely on the financial organization [3]. Powell et al. emphasized that if an enterprise wants to improve its competitiveness, it must fully exploit its own advantages and devote its limited resources to its core business [4]. Kobrak stated that shared service is the first step in financial transformation, and financial shared service is an excellent practice of shared service, and he created a detailed and feasible optimization design for the organizational framework, process, financial information system, operation management, specific implementation, and other aspects of financial shared service [5]. Rau pioneered data warehouse technology in the banking industry as the primary tool for centralized data management [6]. Liu improved the BP neural network (BP neural network) and used it to predict financial distress, with good results [7]. Kirk et al. found that the data had an ideal effect of 89.16% using a nonlinear combination model of fuzzy neural networks [8].

Data in financial accounting can be automatically identified under AI conditions, accounting information can be efficiently recorded and reflected, and data can be analyzed with the help of a network and processing programme, which is more timely than traditional financial accounting. In the AI era, the accounting department should not only do a good job accounting monetary funds but also record relevant data resources based on the data of other companies in order to exert greater labour value and make greater contributions to the company. Although financial exchanges' accounts receivable business has been continuously optimized in line with the trend in recent years, issues such as too many manual operations, long working hours, low invoicing efficiency, and uncontrollable errors have not been adequately addressed. Despite its powerful functions in data calculation, reasoning, and pattern recognition, the intelligent prediction model lacks stability. This article discusses the specific steps in the transformation process by analyzing the differences between management accounting and traditional financial accounting.

### 1.1. Contribution of the Subject

1. Based on the existing information system, this article designs an optimization framework of accounts receivable management based on AI, describes the application of BPNN in accounts receivable risk management in detail, analyzes the optimization contents, and puts forward concrete implementation suggestions from three aspects of financial team transformation.
2. Using a neural network model and combinatorial thinking method, this article constructs the financial distress notification model of listed companies in China. The neural network method can reveal the nonlinear relationship contained in data samples, and a large number of processing units form a nonlinear adaptive dynamic system, which has good adaptability, self-organization, strong learning, relevance, fault tolerance, and capacity, and can flexibly and conveniently model complex unknown coefficients with various reasons.

### 1.2. Organizational Structure of the Article

The first chapter introduces the research background and significance and then introduces the main work of this article. The second chapter mainly introduces the related technologies of financial management. The third chapter puts forward the specific methods and implementation of this research. The fourth chapter verifies the superiority and feasibility of this research model. The fifth chapter is the summary of the full text.

## 2. Related Work

### 2.1. Financial Management Research

To construct the enterprise financial management and decision-making system, the technical route based on theory is to first establish a data warehouse and take the original data collection of the data warehouse as the main task. At present, the original data of the data warehouse established by the survey object come from relevant text information such as enterprise financial information platform.

Basic financial work is an important foundation to improve the level of financial management, covering accounting, system construction, process design, accounting supervision, performance evaluation, and other aspects of management. Dale pointed out that companies with scattered organizational structure or less hierarchical structure can gain management advantages by sharing human resources and technical resources [9]. Byrne defines the essence of a shared service center as an independent organizational entity and emphasizes that the objects of shared services should be molecular companies and business departments within the enterprise [10]. James believed that shared services can integrate all the resources of the company, reduce operating costs, and provide high-quality and professional services for different regions and internal partners, so as to realize enterprise value [11]. Luca and Meschieri believed that in the case of organizational failure, enterprises can gather many basic businesses in a semimarket organizational entity [12]. Tinoco et al. explained the mode of financial sharing service mainly from three aspects: entry management, outflow management, and comprehensive control [13].

### 2.2. Research on Early Warning of Financial Distress

At present, China has not yet established a complete financial early-warning system, and only some financial forecast information is disclosed in the financial statements, which is only a simple extension of the historical and present financial trends. The artificial neural network is a parallel distributed pattern processing system developed from the research results of neuropsychology and cognitive science by applying mathematical methods.

Huang et al. used multivariate discriminant analysis to discuss the prediction of financial distress of enterprises [14]. Gudmunson et al. put forward that the early-warning system is mainly composed of three subsystems: evaluation, warning, and response, in which evaluation is the evaluation and prediction of the existing alarm situation [15]; warning is to transform the forecast of the previous step into information and communicate with each department. Besancenot and Vranceanu used multivariate discriminant analysis to study the early warning of the financial crisis and adopted 22 financial ratios to establish the famous 5-variable Z-Score model through mathematical statistics screening. According to the discriminant score, the financial crisis of the research object was discriminated with a certain critical value [16]. The results of Olafsson's research show that factors such as enterprise size, financial structure, operating performance, and liquidity are highly correlated with the probability of financial distress [17]. Li et al. synthesized the academic definition of financial distress and divided it into four situations: failure, insolvency, default, and bankruptcy [18].

The financial early-warning system is an organizational operation system for analysis, early warning, monitoring, and implementation, which is established to prevent business errors. It adopts the information management method, selects appropriate early-warning indicators according to various financial analysis data, makes a comprehensive and systematic analysis of the company's related financial indicators, and timely informs the company's management about the risks of the company's financial operation system and other stakeholders, so as to take early action to prevent losses.

## 3. Methodology

### 3.1. Optimization of Financial Management Process Based on AI

In the process of transition from traditional accounting to management accounting, it is necessary to start with accountants' own quality, combine the advanced ideas of management accounting with the actual situation, and make good use of AI to cultivate a set of management accounting that can meet the needs of the new era. Accounting can use AI financial system to fully understand the company's cost management information, understand the linkage of cost differences through data analysis, and finally realize effective cost control through problem rectification. In order to make greater contributions, accountants must constantly improve their professional ability, expand their knowledge, and enhance their comprehensive ability to adapt to the development and progress of the times.

In the aspect of accounts receivable management, the main risks faced by enterprises include margin risk and settlement risk, which involve both owner's credit management and accounts receivable risk management. The original documents are mainly composed of manual documents and electronic documents, and the business departments of subsidiaries sort out the original invoices generated by various businesses. Through bill image scanning technology [19, 20], the original bill is converted into image data and stored in the database. Regarding the risk management of accounts receivable, as the damage of overdue accounts receivable to the company is directly reflected in bad debt risk, which damages the company's operating profit, the company should evaluate the risk of accounts receivable in time and guide the business personnel to choose an appropriate payment management method according to the evaluation results.

**Figure 1:**
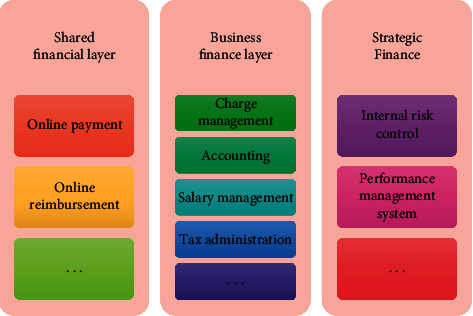
Organizational structure of enterprise financial sharing platform.

Relying on information technology such as cloud computing, financial services scattered in various departments with high repeatability, high turnover rate, and high standardization will be integrated into a new business service platform so as to realize process reengineering and standardization and provide users with quality products. According to the analysis in Figure 1, this document adopts “basic finance, financial management and strategic finance.” Furthermore, the first step of building the platform is completed in collaboration with various departments of the company.

Most employees and users have access to financial information, which primarily includes online payment and reimbursement. The settlement and financial accounting modules, as well as the treasury payment system and banking system, are all linked; the budget management module is linked to the financial department to ensure one-to-one budget item correspondence. It will promote the most profound change in enterprise financial management and provide comprehensive and effective decision support for management while ensuring the efficient operation of the enterprise financial communication platform. Platform business process strategy should be in line with platform strategy. Business process strategy is a shared platform strategy, and business process goal is a shared platform goal, which guides business process optimization and design. After the auditor confirms the original paper receipt, the system will automatically generate the electronic accounting receipt, print the paper version, and begin the electronic payment programme. If the audit fails, the data will be returned in the same manner, with an e-mail sent to remind the refund staff to make the necessary changes.

Accounts receivable have high liquidity and high risk, and their safety and quality will have a significant impact on the company's resources, excess losses, cash flow, etc. and have an important impact on improving the business level of enterprises. Because of its strong fault-tolerant ability, BPNN can ensure that the accuracy of its training results remains at a high level. Assume that the number of nodes in the input layer of BPNN is *c*, the number of nodes in the output layer is *d*, the number of nodes in the hidden layer is *e*, the weights between the hidden layer and the input layer are *V*_*iw*_, and the weights between the hidden layer and the output layer are *V*_*jw*_. Let the hidden layer function be *f*_1_ and the output layer function be *f*_2_.

Among them, the output of the hidden layer node is as follows:

(1) $$\begin{array}{c} Z_{k}=f_{1}\left(\sum_{i=0}^{n}V_{ik}X_{i}\right),\;k=1,2,\ldots ,q. \end{array}$$

For *P* sample sizes, the overall error value is as follows:

(2) $$\begin{array}{c} E=\frac{1}{2}\sum_{P=1}^{P}\sum_{j=1}^{m}{\left(t_{j}^{p}-y_{j}^{p}\right)}^{2}. \end{array}$$

Accounts receivable system is a part of the financial management system, which can work independently or cooperate with other subsystems to transmit relevant data and vouchers. The relationship between the credit system and other subsystems of listed companies' financial participation system mainly includes the following four aspects:

(1) The relationship between A/R management and sales management: A/R management and sales management share A/R balance information, and sales invoices issued by sales management will be fed back to the A/R system in a timely manner; (2) relationship between A/R management and A/P management: write-off of A/R and A/P is supported; (3) relationship between collection management and budget management: budget revenue and expenditure items can be entered in A/R management; (4) relationship between A/R management and fund management: the collection document can generate the fund system collection notice.

**Figure 2:**
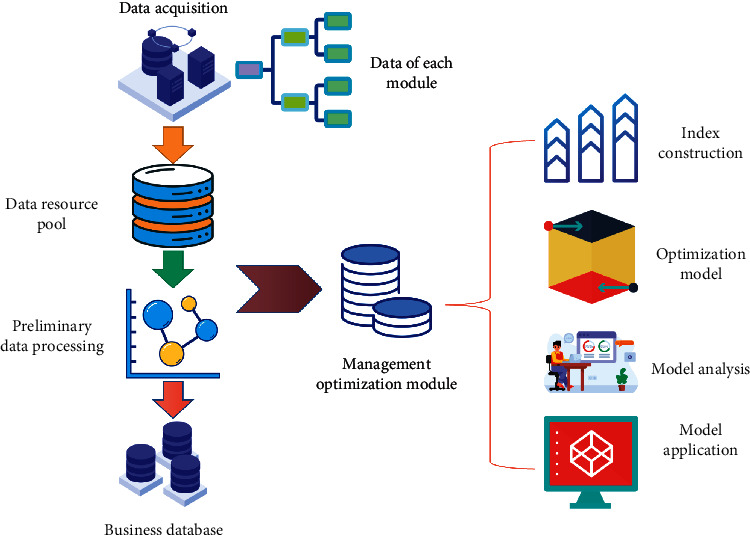
AI-based optimization framework for accounts receivable management of listed companies.

Based on the BPNN default risk prediction model, the automation of finance improves the efficiency of the clearing house and automates the processes with a high repetition rate but is prone to problems, such as invoicing, accounts receivable reconciliation, and collection cancellation, and theses processes have been optimized and improved. The optimization framework of accounts receivable management of listed companies is shown in Figure 2.

DT (decision tree) is an important classification technology [21, 22] in data mining. Its main function is to classify and process the data in the existing training set according to the attributes and class labels of the training dataset and to establish a model according to the algorithm and use it to classify new data. The core content of this algorithm is the process of constructing the DT model. Firstly, the data samples of the training set are analyzed, and then DT is constructed. The DT model is established to analyze the data and then predict and analyze the data.

An improved algorithm of DT algorithm is proposed: DT is actually an improved DT that combines a linear classifier and DT classifier. DT construction method is used to reduce the number of layers in the tree. This method is a measure of DT, which can better improve the efficiency of DT classification. The algorithm used is a supervised algorithm, in which the labeled classification training set is performed in advance.

Compared with the obvious Mahalanobis distance measurement method, all linear methods have the invariance of scale transformation. Let *y*=*Ax*, then, the vector be *x*_1_, *x*_2_, *m*_*x*_, and the transformed distance between them be *y*_1_, *y*_2_, *m*_*y*_. Mahalanobis distance is as follows:

(3) $$\begin{array}{c} {\left\|x-m\right\|}_{M}={\left(x-m\right)}^{T}C^{-1}\left(x-m\right). \end{array}$$

Under the Mahalanobis distance scale, this method provides the basis for distance judgment.

**Figure 3:**
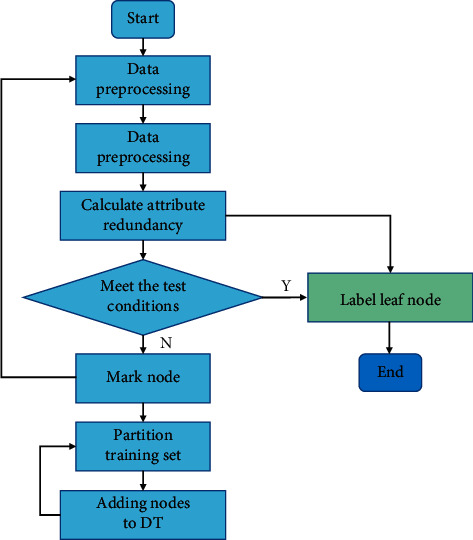
Improve algorithm flow.

This algorithm introduces the concept of measurement method when calculating information drop gain, which can be used as a reference when selecting and classifying. The following Figure 3 shows the process of improving the algorithm.

1. For a superset *C* ∈ *T*, *C* contains several categories of samples. They will wait until the next level to continue the classification.
2. Choosing the best case for classification is the classification method adopted when no attribute set can be found.
3. At the end of the process, all samples are classified or the sample classification cannot be continued.

### 3.2. Research on Financial Distress Prediction

The application and advancement of AI technology has gradually altered the traditional financial accounting function. Accounting work is no longer a single data record, and more financial accountants are needed to effectively organize and extract data. Accounting is an organization's internal accounting, and it focuses on providing information to help management make informed decisions. Financial accounting must be prepared according to strict accounting standards, and management is more flexible than financial accounting. AI can help businesses process financial data more quickly and efficiently and guide them through internal production processes. It can also serve as a reference for managers making predictive decisions. The transition from financial to management accounting is accelerated by AI.

When developing accounting functions, businesses are paying more attention to the integration of other departments and financial departments. As a result, when financial accounting becomes management accounting, the financial department's personnel must meet higher standards. We must start with the company's information construction when transitioning from financial accounting to management accounting. The old enterprise information system equipment should be replaced as soon as possible, and the entire system should be properly optimized to meet intelligence demand. With the advancement of information globalisation, the demand for the transition from financial accounting to management accounting is becoming more apparent, and the trend of enterprise and finance integration is becoming more apparent, which is more conducive to the innovation of the financial management mode of enterprises and increases market share. The unexpected occurrence of financial distress determines the uncertainty of financial distress management. Financial distress is influenced by a variety of factors, some of which can be understood and controlled and others of which are explosive and unexpected. With the advancement of economic globalisation, an increasing number of businesses rely solely on traditional management methods, leaving them unable to plan for rapidly changing activities and market dynamics, and deal with a variety of new problems. More importantly, business leaders are paying increasing attention to the prevention and management of financial difficulties and financial early warning.

Up to now, most of the samples used to test the prediction efficiency of the model have used paired samples, and there is no clear sample pairing rule. As the number of companies in financial distress is very small, the most diversified self-test models can always be found in a large number of nonfinancial distress companies, so the universality of the models is questionable. From the perspective of the neural network mapping relationship, the variable selection method based on the neural network not only avoids the practical difficulty of how to set the shape of function correctly in the modeling process but also expands the function types in regression modeling. Research that makes variable selection research more common and opens up an additional way for variable selection when the structure of the response function is unknown. Choosing forecast variables according to economic and financial theories can make up for this.

Theoretically, three-layer BPNN can approximate any function. In this article, we use a three-layer BPNN with only one hidden layer. The weight is adjusted according to the general gradient descent method: *i* represents the input layer, *j* represents the hidden layer, *k* represents the output layer, and *l* represents the learning step; *w*_*ij*_, *w*_*jk*_ represents the weights of input to the hidden layer and hidden layer to output layer, respectively; *E*(*n*) is the sum of error energies.

(4) $$\begin{array}{c} \Delta w_{ij}\left(n\right)=-\frac{\partial E\left(n\right)}{\partial w_{ij}\left(n\right)},\\ \Delta w_{jk}\left(n\right)=-\frac{\partial E\left(n\right)}{\partial w_{jk}\left(n\right)}. \end{array}$$

**Figure 4:**
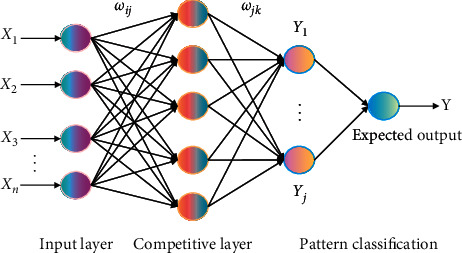
Schematic diagram of BPNN working principle.

Here, we combine dynamic principal component analysis with BPNN to construct a BP prediction model, as shown in Figure 4.

Assuming that the ensemble is composed of *N* independent neural network classifiers, adopting the voting method of an absolute majority, and assuming that each network gives the correct classification result with the probability of 1 − *p*, the errors among the networks are uncorrelated and the probability of errors in the ensemble neural network is *P*_error_:

(5) $$\begin{array}{c} P_{\text{error}}=\sum_{k>N/2}^{N}\left(\begin{array}{c} N\\ k \end{array}\right)p^{k}{\left(1-p\right)}^{N-k}. \end{array}$$

At *p* < 1/2, *P*_error_ decreases monotonically with the increase of *N*. Therefore, if the prediction accuracy of each neural network is higher than 50%, and the errors among the networks are uncorrelated, the more the networks in neural network integration, the higher the integration accuracy. When *N* tends to infinity, the error rate of integration tends to zero.

Aiming at these shortcomings of the BPNN algorithm, this paper improves the original BPNN algorithm. The adaptive learning method and momentum summation method are used to modify the standard BPNN algorithm, which can effectively avoid the problem that the network falls into local minima. The artificial neural network can be mapped in any form, and financial early warning provides a new way of thinking.

The concrete improvement method of the BPNN algorithm is shown as follows.

In fact, the traditional BPNN algorithm is a simple high-speed descending static optimization algorithm. When correcting the weight *w*(*k*+1), it is only corrected according to the negative gradient direction at time *k*, without considering the previous accumulated experience. Its weight correction function is written as follows:

(6) $$\begin{array}{c} w\left(k+1\right)=w\left(k\right)+\eta \left(k\right), \end{array}$$

where the weight of *w*(*k*) connection is the negative gradient of *k* moment:

(7) $$\begin{array}{c} D\left(k\right)=\frac{-\partial V}{\partial w\left(k\right)}, \end{array}$$

where *V* is the square error between the actual output and the desired output of the network, and *η* is the learning rate.

A good learning rate at the beginning of training may not be suitable for later training. In order to solve this problem, it is natural to think of automatically adjusting the learning rate during the training process, so an improved algorithm of self-adaptive adjusting the learning rate appears, and its calculation formula is as follows:

(8) $$\begin{array}{c} w\left(k+1\right)=w\left(k\right)+\eta \left(k\right)D\left(k\right),\\ \eta \left(k\right)=2^{\lambda }\eta \left(k-1\right). \end{array}$$

It can be seen that when the gradient direction of two successive iterations is the same, it means that the descent is too slow and the step size can be doubled; when the gradient directions of two successive iterations are opposite, it means that the descent time is too long, and the step size can be halved. Therefore, the network convergence can be accelerated by constantly adjusting the learning rate.

## 4. Experiment and Results

In order to analyze the progress of the company's financial budget implementation and the past years, it is necessary to extract the past years' budget data from the financial data warehouse and achieve the purpose by data aggregation and other calculation methods. The implementation of intelligent decision-making in financial budget management is divided into two steps:

1. For analysis and application, firstly, intelligent decision-making based on the company's financial data warehouse and multidimensional analysis is established;
2. Data mining is used to perform intelligent decision-making function.

The calculation of information gain depends on the attributes with more values, and the information gain depends on the number of attribute values, but in fact, this choice is meaningless in some cases. For example, the identification number is the identification attribute of a piece of data, and the identification attribute is the unique identification of the corresponding information. As the data value becomes larger and larger, the information gain corresponding to the port attribute is finally maximized.

In the process of DT construction, the key step is the splitting of nodes. Algorithm C4.5 uses information gain rate to measure the advantage of split nodes. In the formula of information entropy and information gain, the ratio of information gain is information gain and information entropy.

**Figure 5:**
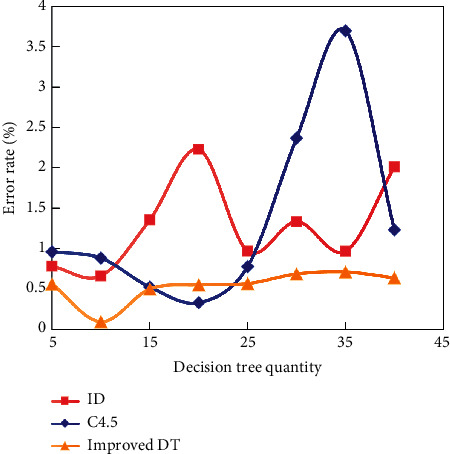
Comparison between DT constructed by the improved algorithm and traditional DT.

ID3, C4.5, and improved DT algorithms are run on different datasets, and the experimental data are obtained. The improved DT algorithm, ID3 algorithm, and C4.5 algorithm are compared, respectively, and the classification performance of the improved DT algorithm is compared. The error rate of the improved DT algorithm is significantly higher than that of the ID3 algorithm and C4.5 algorithm. The DT constructed by the improved algorithm is compared with the DT constructed by the traditional algorithm ID3 and C4.5, as shown in Figure 5.

The improved DT produces three layers of DT, according to the experimental results. Only one dataset is misclassified in each classification structure. When you examine the misclassified data, you will notice that each characteristic value is very similar to the misclassified class, not its own. The accuracy of the improved algorithm has improved. There is no guarantee that the final results will be the best, and the relationship between them is not linear. The tree hierarchy can be reduced effectively in order to improve the classification result.

The focus of accounts receivable analysis should be on the liquidity analysis of accounts receivable. Through trend analysis and structure analysis, we can find out the changing rules of accounts receivable, then pay attention to accounts receivable in abnormal situations, and analyze the economic essence that causes abnormal changes. The *p* value of the Bartlett spherical test is close to 0, which indicates that there is a linear correlation between indicators, which is suitable for factor analysis. When the factor reaches the fourth place, the cumulative variance contribution rate is 95.32%, indicating that almost all the information of the original variable is retained. At this time, the number of factors is only 2/3 of the original, thus achieving the purpose of simplifying the indicators. Therefore, this article selects three factors for analysis, and the total explanatory variance is shown in Table 1.

Firstly, according to the comprehensive score of the owner unit, this article classifies the accounts receivable risk of the owner unit in the model sample and then finds out the dividing points of each level. The cluster analysis results are shown in Table 2.

After obtaining the evaluation results, carry out a risk assessment and select the appropriate collection strategy. Overdue accounts collection is the joint responsibility of the subsidiary business department and the legal department, and the legal department takes the lead in cooperation with the business department. Through various channels, we can fully and truly understand the reasons why the owner cannot pay the project payment on time. And the legal department of the business department shall adopt appropriate nodes to collect according to the split result and collection policy.

**Figure 6:**
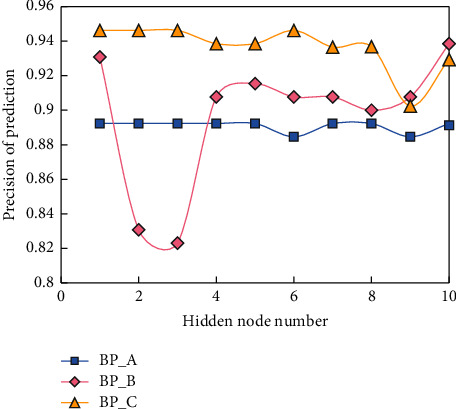
Empirical results of BP model.

In order to easily reflect the role of dynamic principal component analysis in financial forecasting, we conducted three groups of experiments on three models. The first model: the original data are directly predicted by BPNN without any processing (called BP_A). Model 2: Firstly, the data are standardized to eliminate the industry differences, then the original data are processed by static PCA, and a new variable combination (called BP_B) is obtained. The third model is the same: the industry differences are corrected; first, the original data are processed by dynamic principal component analysis, and then the prediction is made by BPNN (called BP_C). The empirical results of the BP model are shown in Figure 6.

It can be seen from the experimental results that there is no difference between BP_B and BP_C, so there is no difference between their results. Compared with the BP_A model, the prediction accuracy is not greatly improved, but the stability is improved. However, because the BP_C model increases the amount of information without increasing the network input and the complexity of the network structure, it not only improves the accuracy but also keeps the stability. When using three-year data, although the complexity of the BP_A model is reduced and the accuracy is improved, it is still not as accurate as when using one-year data. Compared with BP_A, BP_B itself greatly reduces the input dimension, and its accuracy is also in the BP_C model. The input dimension and network complexity do not increase, which shows that the data have little impact on the business.

**Figure 7:**
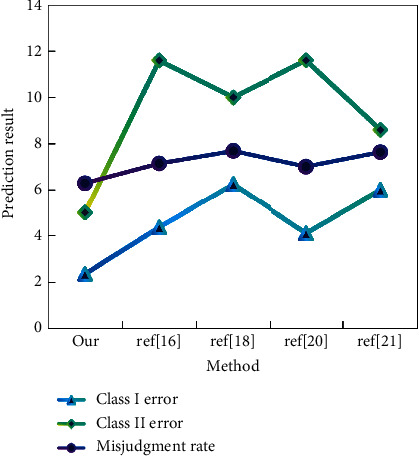
Detailed empirical results of three groups of data.

Here, we combine the dynamic principal component analysis with two network models to obtain two prediction models. In these two models, we select the single bound function and its first, second, and third derivatives to form the mapping function of network nodes. For the output node, we choose a single limiting function. For empirical data, we still use the three groups used above. The detailed results of the three datasets are shown in Figure 7.

**Figure 8:**
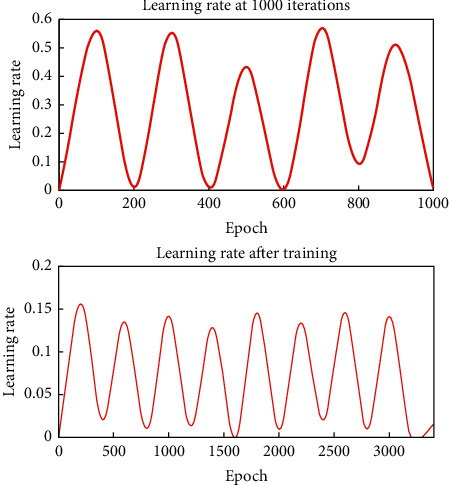
Learning rate curve after the training of financial distress early warning model.

Judging from the prediction results, compared with other types, the prediction accuracy of BPNN has been significantly improved, especially after using data for many years; the prediction results have reached more than 90%, which is unmatched by other methods. Theoretically speaking, dynamic principal component analysis is used to select the variables of financial distress prediction, and an integrated network is used to predict the financial distress of listed companies, all of which have achieved ideal results. MATLAB is used to analyze and design the neural network system. First, a new M-File is created, and then a program is written in the editing bar. Because the input of this network model is a continuous variable, the premnmx function of MATLAB is used to normalize the training and unify the network output before processing. The learning rate curve of this network is shown in Figure 8.

**Figure 9:**
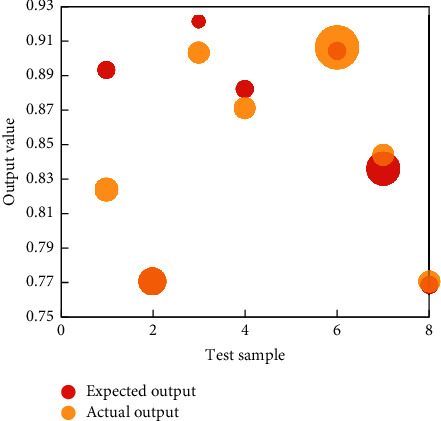
Judgment result of the neural model test.

Then the test sample is used for testing. See Figure 9 for the training and testing results.

The empirical research on the training, testing, and evaluation of the financial early-warning method model established in this article shows that the established neural network model has a good prediction effect and the actual results of the sample are close to the expected results, which are quite consistent, and its financial early-warning results are completely in line with its financial situation, which also shows that the application of BPNN in FinTech has a good prediction effect. On the other hand, the use of the BPNN algorithm and network topology configuration can be studied further, and a neural network and expert system combination can be used to see if the ability to predict financial difficulties can be improved. Naturally, this model has flaws, one of which is the number of nodes in the hidden layer. Constant training and sample testing are required to determine the number of nodes, and once convergence is achieved, the number of nodes meets the requirements. This procedure necessitates a significant amount of effort.

## 5. Conclusions

The shift from accounting to management accounting is a general trend that is unavoidable, given the new era's economic situation and the industry's long-term development. The role of enterprise accounting is changing, and the transition from financial to management accounting is speeding up, thanks to the advancement of modern information technology in the financial field. At the same time, management accounting is becoming more computerised. The government and other relevant departments must supervise the company internally. This document uses AI technology as the foundation for optimizing accounts receivable management in the integration mode and adopts a financial management optimization design based on AI to meet listed companies' accounts receivable management needs. The financial distress early-warning model of publicly traded companies is built using the BP neural network method. The empirical results show that, when compared to other types, BPNN has a significantly higher prediction accuracy. It has the potential to help operators take effective actions early in the financial crisis, improve their operating conditions, and avoid business failures.

## Figures and Tables

**Table 1:** Total variance of interpretation.

Component part	Total	Variance percentage	Cumulative%
1	2.201	36.252	36.252
2	1.936	30.287	68.632
3	1.002	15.642	85.241
4	0.425	8.869	93.368
5	0.225	4.213	99.012

**Table 2:** Cluster analysis results.

Central point	Sample size
−12.363	3
72.541	20
−69.283	1
30.325	14
−28.736	5

## Data Availability

The data used to support the findings of this study are available from the author upon request.

## References

[1] Huang Y., Cheng L., Xue L. (2021). Deep adversarial imitation reinforcement learning for QoS-aware cloud job scheduling. *IEEE Systems Journal*.

[2] Chen J., Du C., Zhang Y., Han P., Wei W. (2021). A clustering-based coverage path planning method for autonomous heterogeneous UAVs. *IEEE Transactions on Intelligent Transportation Systems*.

[3] Eckles D. L., Hoyt R. E., Miller S. M. (2014). Reprint of: the impact of enterprise risk management on the marginal cost of reducing risk: evidence from the insurance industry. *Journal of Banking & Finance*.

[4] Powell M., Gillett A., Doherty B. (2018). Sustainability in social enterprise: hybrid organizing in public services. *Public Management Review*.

[5] Kobrak C. (2009). Family finance: value creation and the democratization of cross-border governance. *Enterprise and Society*.

[6] Hu S., Yu B. (2020). *Big Data Analytics for Cyber-Physical Systems*.

[7] Liu C. (2011). Failures of enterprise-level unionization in China: implications for coalmine safety and beyond. *International Labour Review*.

[8] Kirk G., Beth Nolan S. (2010). Nonprofit mission statement focus and financial performance. *Nonprofit Management and Leadership*.

[9] Bell A. R., Dale R. S. (2011). The medieval pilgrimage business. *Enterprise and Society*.

[10] Byrne T. (2009). Debating enterprise social software. *EContent*.

[11] James J. (2009). Plight of the fortune tellers: why we need to manage financial risk differently. *Financial Analysts Journal*.

[12] Luca F. D. e., Meschieri E. (2017). Financial distress pre-warning indicators: a case study on Italian listed companies. *The Journal of Credit Risk*.

[13] Tinoco M. H., Holmes P., Wilson N. (2018). Polytomous response financial distress models: the role of accounting, market and macroeconomic variables - ScienceDirect. *International Review of Financial Analysis*.

[14] Huang J., Wang H., Kochenberger G. (2017). Distressed Chinese firm prediction with discretized data. *Management Decision*.

[15] Gudmunson C. G., Son S., Lee J., Bauer J. W. (2010). EITC participation and association with financial distress among rural low-income families. *Family Relations*.

[16] Besancenot D., Vranceanu R. (2008). Financial distress and banks’ communication policy in crisis times. *Romanian journal of economic forecasting*.

[17] Olafsson A. (2016). Household financial distress and initial endowments: evidence from the 2008 financial crisis. *Health Economics*.

[18] Li S., Shi W., Wang J., Zhou H. (2021). A deep learning-based approach to constructing a domain sentiment lexicon: a case study in financial distress prediction. *Information Processing & Management*.

[19] Zhou J., Wei X., Shi J., Chu W., Zhang W. (2022). Underwater image enhancement method with light scattering characteristics. *Computers & Electrical Engineering*.

[20] Zhou J., Liu D., Xie X., Zhang W. (2022). Underwater image restoration by red channel compensation and underwater median dark channel prior. *Applied Optics*.

[21] Cai W., Wei Z. (2022). Remote sensing image classification based on a cross-attention mechanism and graph convolution. *IEEE Geoscience and Remote Sensing Letters*.

[22] Zhang J., Wang W., Lu C., Wang J., Sangaiah A. K. (2020). Lightweight deep network for traffic sign classification. *Annals of Telecommunications*.

